# The Relation Between Inflation in Type-I and Type-II Error Rate and Population Divergence in Genome-Wide Association Analysis of Multi-Ethnic Populations

**DOI:** 10.1007/s10519-017-9837-3

**Published:** 2017-02-10

**Authors:** E. M. Derks, A. H. Zwinderman, E. R. Gamazon

**Affiliations:** 1 Department of Psychiatry, Academic Medical Center, University of Amsterdam, Meibergdreef 9, 1105 AZ Amsterdam, The Netherlands; 2 QIMR Berghofer, Translational Neurogenomics group, Brisbane, Australia; 3 Department of Clinical Epidemiology and Bio-Statistics, Academic Medical Center, University of Amsterdam, Meibergdreef 9, 1105 AZ Amsterdam, The Netherlands; 4 Division of Genetic Medicine, Department of Medicine, Vanderbilt University, 2215 Garland Avenue, Nashville, TN USA

**Keywords:** Genome Wide Association, GWAS, Population stratification, Population divergence, F_ST_, Admixture, Principal components

## Abstract

Population divergence impacts the degree of population stratification in Genome Wide Association Studies. We aim to: (i) investigate type-I error rate as a function of population divergence (F_ST_) in multi-ethnic (admixed) populations; (ii) evaluate the statistical power and effect size estimates; and (iii) investigate the impact of population stratification on the results of gene-based analyses. Quantitative phenotypes were simulated. Type-I error rate was investigated for Single Nucleotide Polymorphisms (SNPs) with varying levels of F_ST_ between the ancestral European and African populations. Type-II error rate was investigated for a SNP characterized by a high value of F_ST_. In all tests, genomic MDS components were included to correct for population stratification. Type-I and type-II error rate was adequately controlled in a population that included two distinct ethnic populations but not in admixed samples. Statistical power was reduced in the admixed samples. Gene-based tests showed no residual inflation in type-I error rate.

## Introduction

Genome-Wide Association Study (GWAS) is a widely used approach for the identification of genetic variants that are associated with disease risk (Manolio 2010). GWAS should carefully control for the potential presence of genetically heterogeneous subgroups within the study, e.g., samples with different ethnic backgrounds (Price et al. 2006; Stringer et al. 2015), since unequal distribution of patients and controls among ethnic groups may result in spurious genetic associations. Therefore, the most widely used approach is to conduct GWAS within a genetically homogeneous group. However, as pointed out by Medina-Gomez and colleagues, excluding subjects with different ethnic backgrounds may reduce sample size and thereby lead to a loss of statistical power (Medina-Gomez et al. 2015).

For these reasons, Medina-Gomez et al. aimed to investigate the effect of population admixture on type-I error rate in Genome Wide Association Studies (GWAS) (Medina-Gomez et al. 2015). The authors analyzed two example phenotypes: red hair pigmentation; a binary trait characterized by large stratification across ethnic populations and skull bone mineral density (skull BMD); a quantitative phenotype. Correction for population stratification was done by including genomic components as covariates in the association model (see, e.g., (Price et al. 2006) or alternatively, by using Efficient Mixed-Model Association eXpedited (EMMAX) analysis (Kang et al. 2010). Based on the results of their analyses, the authors concluded that spurious associations in admixed samples can be handled adequately.

However, several limitations may prevent generalization of these findings. First, because the authors analyzed observed phenotypic data for which true genetic effect sizes are unknown, their analyses do not allow firm conclusions on type-I error rate and bias in effect size estimation. Second, the relation between strength of population divergence and inflation of test statistics has not been investigated, even though this is important since inflation may be limited to those Single Nucleotide Polymorphisms (SNPs) which show substantially different allele frequencies across ethnic groups. The level of population divergence, also referred to as the level of fixation index (F_ST_), is a measure of population differentiation due to population structure. The F_ST_ is calculated using information on the frequency of genetic polymorphisms across populations. Third, the authors focused primarily on controlling the type-I error rate and did not investigate the impact on the statistical power to detect true associations. Finally, no gene-based analyses were performed. Gene-based tests investigate whether a single gene contains multiple variants that contribute independently to a trait. A previous study showed that population stratification has a particularly strong impact on gene-based tests of low-frequency genetic variants (Liu et al. 2013) which warrants a further investigation of the performance of gene-based tests.

It is surprising that the impact of population divergence (F_ST_) on genetic association tests has not been systematically examined. Price et al. (2006) previously investigated, in SNP-based GWAS, the rate of false positive associations and the power to detect true associations for a correction that utilizes principal components and for an approach that applies uniform adjustment by genomic control (Devlin and Roeder 1999). However, for random SNPs with no association to disease (i.e., for type-I error analysis) and for causal SNPs (i.e., for power analysis), they modeled allele frequency differences (i.e., F_ST_) using the Balding-Nichols method (Balding and Nichols 1995) and assumed that population differentiation was equal for all genetic variants (F_ST_ = 0.01); furthermore, for differentiated SNPs with no association to disease, they assumed allele frequencies of 0.80 and 0.20 for the two ancestral populations. Our study investigates the generalizability of their findings to the *empirically observed* genome-wide distribution of allele frequency differences between ancestral populations from subsequent large-scale genotyping and sequencing of human populations (Altshuler et al. 2010; Auton et al. 2015). In addition, although they applied a principal component based approach on real GWAS data, the samples used were of a relatively homogeneous European ancestry. Especially in samples with multiple source populations, although continuous axes of variation often have a geographic interpretation (Novembre et al. 2008), the number of axes included in the adjustment is often not so easily biologically interpretable (with some axes possibly representing sampling or technical artifacts) but the number of included axes may indeed substantially influence the resulting type-I error rate and statistical power. The relevance of these axes of variation is also an important question for studies with (admixed) samples [such as considered here (Auton et al. 2015)] in which the local ancestry variability differs substantially from the global ancestry variability.

We extend the work of Medina-Gomez and colleagues by conducting a simulation study in which phenotypic data are generated in the European (CEU) and African (YRI) populations of the “Hapmap” (Altshuler et al. 2010) data and in the CEU and Americans of African Ancestry in SouthWest USA (ASW) samples of the “1KG” (Auton et al. 2015) data. The Hapmap CEU and YRI samples were used to simulate population stratification due to two genetically distinct but relatively homogeneous populations while the 1KG ASW, characterized by a relatively high level of admixture, and CEU samples were used to simulate a more complex form of population stratification. Phenotypes were simulated according to genetic models for which the model parameters were set at desired values. In our simulations, we considered the important example of two source populations showing substantial trait divergence (e.g., differential disease susceptibility or health disparity). The aims of this study are: (i) to investigate whether type-I error rate in multi-ethnic populations can be controlled for genetic variants with varying levels of population divergence; (ii) to evaluate the degree to which correcting for population stratification reduces statistical power or leads to biased effect size estimates; (iii) to investigate the impact of population stratification on the results of gene-based analyses.

## Methods

### Genotypic data

Genotypes of publicly available Hapmap draft release 3 data (http://hapmap.ncbi.nlm.nih.gov/downloads/genotypes/2010-05_phaseIII/plink_format/) (Altshuler et al. 2010) and 1KG data (ftp://ftp.1000genomes.ebi.ac.uk/vol1/ftp/release/20110521) (Auton et al. 2015) were used for the simulation analyses. In the Hapmap sample, analyses were restricted to subjects with Northern or Western European background (CEU) or African ancestry (YRI) while in the 1KG data, analyses were restricted to the CEU subjects and Americans of African Ancestry in SouthWest USA (ASW).

### Calculation of F_ST_

For 3,173,374 and 30,102,059 SNPs in the Hapmap and 1KG dataset (respectively), population divergence between the ancestral European and African populations was quantified using the fixation index (F_ST_). We used the Weir and Cockerham (Weir and Cockerham 1984) unbiased estimator of the F_ST_ statistic:

1 $${{F}_{ST}}~=~{}^{\left( MSP~-~MSG \right)}\!\!\diagup\!\!{}_{\left( MSP~+~\left( {{n}_{c}}-1 \right)MSG \right)}\;$$

where,

$${{n}_{c}}=\left( \mathop{\sum }^{}{{n}_{i}}-\frac{\mathop{\sum }^{}{{n}_{i}}^{2}}{\mathop{\sum }^{}{{n}_{i}}} \right)$$

$$MSP=\mathop{\sum }^{}\left( {{n}_{i}}{{\left( {{p}_{i}}-p \right)}^{2}} \right)$$

$$MSG=\left( {}^{1}\!\!\diagup\!\!{}_{\mathop{\sum }^{}\left( {{n}_{i}}-1 \right)}\; \right)\mathop{\sum }^{}{{n}_{i}}{{p}_{i}}\left( 1-{{p}_{i}} \right)$$

*n*_*i*_ is sample size in population *i* (=1, 2), *p*_*i*_ is frequency of the given allele in population *i*, and *p* is the average frequency of the allele across the populations while MSP and MSG denote the population variance and the genetic variance, respectively.

### Data quality checks

Initially, the Hapmap data included 1,397 individuals and 1,457,897 SNPs. Analyses were restricted to 259 founders with CEU and YRI ethnic backgrounds. Next, genotype and individual missingness was controlled by (i) excluding SNPs with high levels of missingness (>0.2); and (ii) excluding individuals and SNPs with missingness >0.05. SNPs with minor allele frequency (MAF) <0.01 were excluded. Finally, analyses were limited to SNPs with well-defined F_ST_ values. These quality checks resulted in a final sample of 259 individuals (112 CEU and 147 YRI) and 1,304,792 SNPs.

The downloaded 1KG vcf chromosome files were transformed into Plink format data and merged into a single binary Plink file. This file included 1,029 individuals and 38,151,414 SNPs. Analyses were restricted to 146 founders with CEU and ASW ethnic backgrounds. The same quality checks were performed as for the Hapmap sample, but as an additional step, variants with one or more multi-character allele codes were excluded with the –snps-only option in Plink. These QC steps resulted in a final sample of 146 individuals (85 CEU and 61 ASW) and 5,131,518 SNPs.

### Calculation of MDS components

Twenty multi-dimensional scaling (MDS) components were calculated in PLINK with the option --cluster --mds-plot 20. The MDS components were included as covariates in the genetic association analyses to correct for population stratification. We initially corrected for 10 components, but the number was increased in the presence of remaining inflation of the test statistics.

### Investigation of type-I error rate

Quantitative phenotypes were simulated for the CEU and YRI samples of the Hapmap data, and for the CEU and ASW samples of the 1KG data, under the assumption of strong population trait divergence (e.g., significant differential trait scores) across ethnic populations. Quantitative phenotypes were drawn from normal distributions with means of 0 and 3, respectively, and standard deviations of 1. To investigate the impact of population stratification on type-I error rate (prior to and after MDS correction), the inflation in test statistics and p-values was investigated by calculating the average and standard error of the genomic inflation factor based on 100 simulations. Quantile–quantile (QQ) plots of observed and expected p-values were created for one of these simulations.

Here we consider the mean test statistic (for a quantitative trait) on the non-causal variants to investigate the sources of inflation. Assuming *p* causal variants and *L* total number of non-causal SNPs, the mean ${\chi }^{2}$ statistic on the non-causal variants can be derived (Sham and Purcell 2014) from the non-centrality parameter (NCP) as follows:

2 $${{\lambda }_{mean}}=1+\frac{1}{L}\underset{j=1}{\overset{p}{\mathop \sum }}\,\underset{k=1}{\overset{m\left( j \right)}{\mathop \sum }}\,\frac{Nh_{j}^{2}r_{jk}^{2}}{1-h_{j}^{2}r_{jk}^{2}}$$

Here *N* is the sample size, $h_{j}^{2}$ is the (non-zero) heritability attributable to the causal variant *j*, and $r_{jk}^{2}$ is the linkage disequilibrium between the causal variant *j* and the (non-causal) SNP *k* (Spencer et al. 2009). Note that the NCP for SNP *k* in LD with causal variant *j* decays with LD and is given by $\frac{Nh_{j}^{2}r_{jk}^{2}}{1-h_{j}^{2}r_{jk}^{2}}$. Thus the genomic inflation factor increases with the sample size *N* in a linear manner and may be influenced by longer-range (admixture) linkage disequilibrium. As has been noted, polygenicity [i.e., a large number *p* of contributing variants] may also result in an inflated distribution of the test statistic (Bulik-Sullivan et al. 2015).

### Investigation of statistical power

Quantitative phenotypes were simulated for the CEU and YRI/ASW samples under the assumption of strong population trait divergence for a SNP with a high value of F_ST_. In Hapmap, the selected causal SNP (rs711274) has a F_ST_ value of 0.74. For 1KG, the selected causal SNP (rs7530465) has a F_ST_ value of 0.65. In each analysis, 10,000 simulations were performed. Phenotypes were simulated to be associated with ethnicity and the SNP genotype according to the following model in which ${N}_{j}$ represents the number of risk alleles {0,1,2} for the causal SNP*j*.

$$\text{CEU}:y\sim{}N(0,1)+\text{ SNPeff}*{{N}_{j}}$$

$$\text{YRI/ASW}:y\sim{}N(4,1)+\text{ SNPeff}*{{N}_{j}}$$

The theoretical value of the statistical power was calculated by performing 10,000 simulations in the full samples in the absence of population trait divergence. The effect sizes (SNPeff) in Hapmap and 1KG data were set at 0.2 and 0.3, respectively; a larger effect size was applied in 1KG data to compensate for the smaller sample size. The impact of including the MDS components on statistical power was assessed by repeating the association tests while correcting for the same number of MDS components as in the tests performed in the presence of population trait divergence. This allowed us to quantify the loss in power due to the application of the MDS correction (whether or not there was any difference in phenotype between the source populations) and any additional loss in power from the correction in the presence of population trait divergence.

### Gene-based tests

Finally, we investigated inflation in gene-based tests which may include multiple correlated SNPs and may therefore be more strongly affected by population stratification. We mapped the SNPs to the genomic intervals defined by genes (as defined by GENCODE) (Harrow et al. 2006) using the “intersectBed” command in the bedtools suite (Quinlan and Hall 2010). SNPs which map to multiple genes were excluded. The gene-defined intervals defined the sets on which gene-based tests were performed. For these gene-based tests, analysis was restricted to sets that include at least two variants and were then repeated for genes which include at least five variants to investigate the impact of the number of SNPs in a set on inflation. Gene-based tests were performed with –set-test in Plink; using 10,000 permutations per set and the following options: --set-r2 0.1 --set-p 0.10 --set-max 10.

## Results

### Number of SNPs and distribution of F_ST_

 Hapmap data included 1,304,792 SNPs in total, of which 218,185 (16.7%) showed F_ST_ > 0.25 while 35,303 SNPs showed F_ST_ > 0.5 (2.7%). In the 1KG admixed samples, the total number of SNPs was higher (N = 5,130,324) with similar percentages of SNPs with F_ST_ >0.25 (N = 907,491; 17.7%) and F_ST_ >0.5 (N = 147,891; 2.9%).

### Type-I error rate

**Fig. 1:**
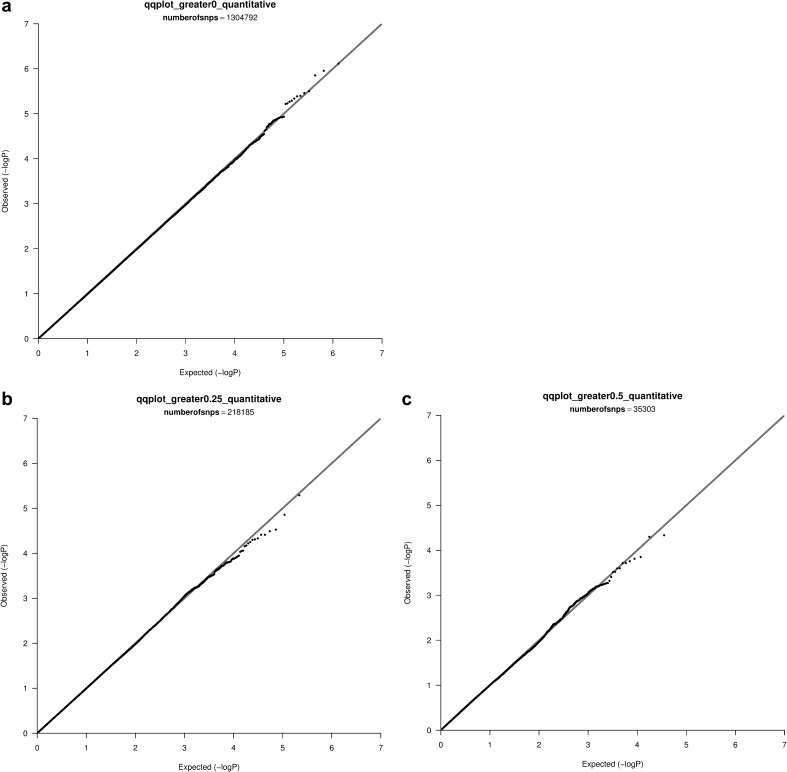
**a** QQ plot, all SNPS, Hapmap data, **b** QQ plot, F_ST_ greater than 0.25, Hapmap data, **c** QQ plot, F_ST_ greater than 0.50, Hapmap data

GWAS analysis of 100 simulations of the Hapmap (“non-admixed”) data did not show evidence of inflation of type-I error rate after adjusting for population stratification using the genomic components. Indeed, after including 10 MDS components, genomic inflation factors were controlled at the expected values of 1 for all SNPs (mean = 1.00, sd = 0.01, p = 1.00), SNPs with F_ST_ >0.25 (mean = 1.00, sd = 0.02, p = 1.00) and SNPs with F_ST_ >0.50 (mean = 1.00, sd = 0.04, p = 1.00). QQ plots for a single simulation are shown in Fig. 1a–c; these plots confirm that the adjustment for population stratification was sufficient to control type-I error rate.

**Fig. 2:**
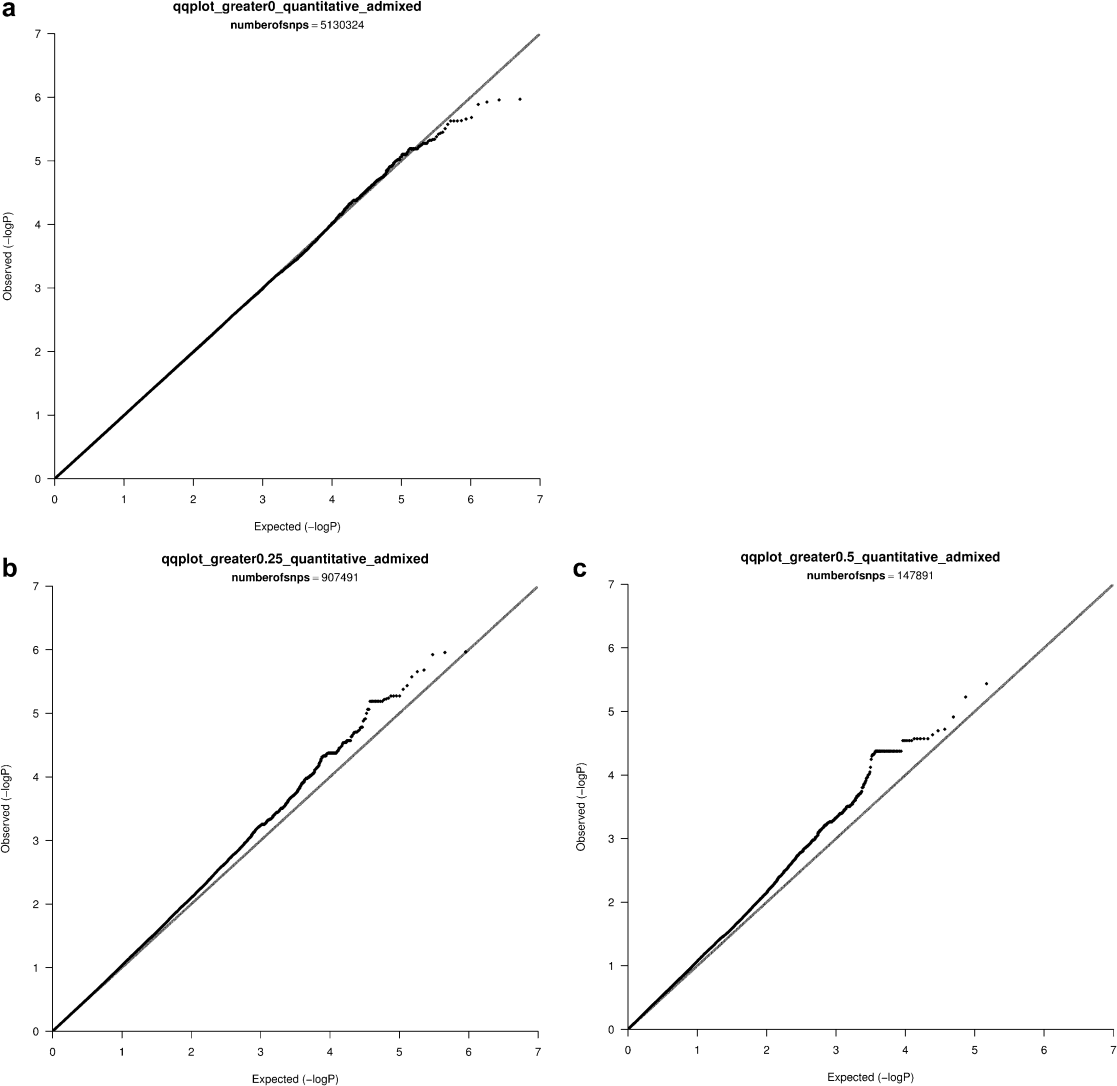
**a** QQ plot, all SNPS, 1KG, **b** QQ plot, F_ST_ greater than 0.25, 1KG data, **c** QQ plot, F_ST_ greater than 0.50, 1KG data

Next, we analyzed the simulated data of the 1KG (“admixed”) samples. Twenty MDS components were included to correct for population stratification. (In these samples, the inclusion of only 10 MDS components, in contrast to the results from the Hapmap samples, was not adequate to correct for the inflation of type-I error rate.) The genomic inflation factor was well controlled when analyzing all SNPs (mean = 1.00, sd = 0.01, p = 1.00), but inflation was observed for SNPs with F_ST_ >0.25 (mean = 1.02, sd = 0.05, p = 0.0001), and for SNPs with F_ST_ >0.5 (mean = 1.04, sd = 0.09, p < 0.0001). QQ plots for a single simulation are shown in Fig. 2a–c. Figure 2b, c show marked inflation, as the observed distribution of p-values deviates from the expected distribution under the null hypothesis. The degree of inflation (as quantified by genomic inflation factor) increased with the degree of SNP population differentiation (as measured by F_ST_).

We subsequently tested whether a mixed linear model leave-one-chromosome-out analysis (such as implemented using the --mlma-loco option in GCTA) would prevent false positive associations. This approach controls for population stratification by taking into account the genetic relationship matrix (which models genome-wide sample structure) defined by all autosomal chromosomes except the chromosome on which the tested SNP is located (Yang et al. 2014). Still, the genomic inflation factors were higher than expected (i.e., averages of 1.06, 1.01, and 1.03 for all SNPs, SNPs with F_ST_ >0.25, and SNPs with F_ST_ >0.50, respectively).

### Effect size estimate and statistical power

Table 1 summarizes the mean estimates of the beta and the statistical power (i.e., the proportion in which the simulation resulted in a p-value <0.05) for rs711274 observed in Hapmap data,. The theoretical power, in the absence of population trait divergence, with no MDS components included as covariates was 0.46. After adjusting for 10 MDS components, the power decreased to 0.39. These estimates were used as reference for the subsequent power analysis conducted in the presence of population trait divergence. When population trait divergence was present, the estimated effect sizes were unbiased (0.20). The statistical power was 0.39, which is similar to the results from the simulations in which no population trait divergence was present. Therefore, the reduction in power was caused by the inclusion of the MDS covariates as correction (with or without population trait divergence) in the context of the relatively small sample size of the Hapmap data.

**Table 1:** Effect size estimates and statistical power from 10,000 simulations

	Hapmap			Admixed 1KG		
	No population trait divergence, No MDS components	No population trait divergence, 10 MDS components	With population trait divergence, 10 MDS components	No population trait divergence, No MDS components	No population trait divergence, 20 MDS components	With population trait divergence, 20 MDS components
Mean beta	0.20	0.20	0.20	0.30	0.30	0.30
Power	0.46	0.39	0.39	0.70	0.36	0.31

In the 1KG data, the effect size estimates were unbiased (see Table 1). However, the power was reduced (0.31) compared to the theoretical value of 0.70. This decrease in power was not completely explained by the inclusion of 20 MDS components as we observed a power of 0.36 when 20 MDS were included in a model with no population trait divergence. Thus, in contrast to the results from the Hapmap samples, the more admixed samples in the 1KG data showed additional loss in power from the correction for population stratification in the presence of population trait divergence.

### Gene-based tests

**Fig. 3:**
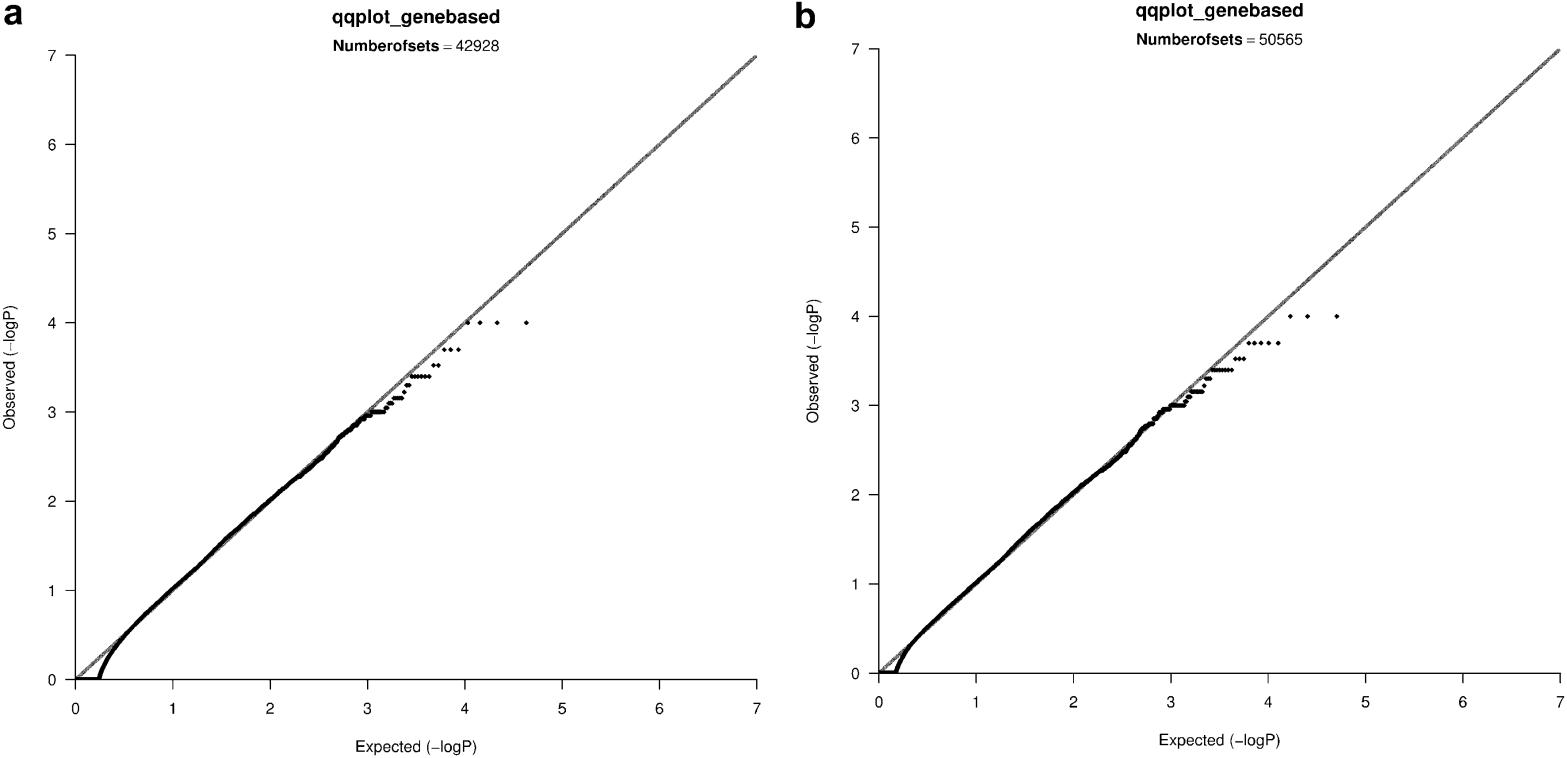
**a** QQ plot of the gene-based tests, sets with ≥2 SNPs, Hapmap, **b** QQ plot of the gene-based tests, sets with ≥5 SNPs, Hapmap

**Fig. 4:**
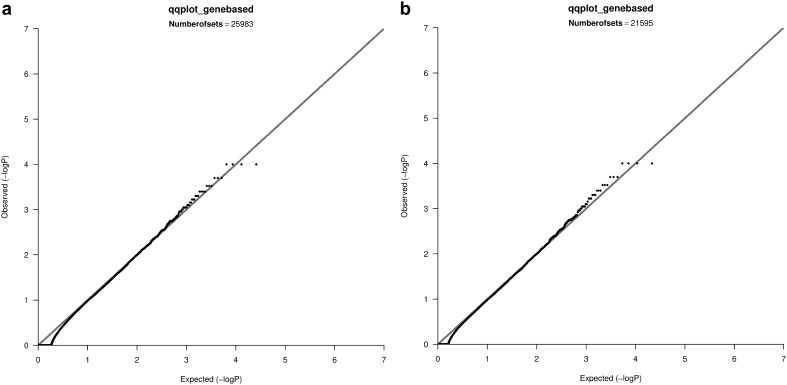
**a** QQ plot of the gene-based tests, sets with ≥2 SNPs, 1KG, **b** QQ plot of the gene-based tests, sets with ≥5 SNPs, 1KG

In the CEU and YRI samples of the Hapmap data, mild inflation was observed when controlling for 10 MDS components (in contrast to the lack of residual inflation from the single-SNP analysis after adjusting for the same number of MDS components in the same samples). Therefore, we repeated the gene-based tests now assuming 20 MDS components. These tests did not show evidence of remaining inflation of type-I error rate (see Fig. 3a, b). Gene-based analyses of the CEU and admixed samples of the 1KG data after adjusting for 20 MDS components did not show residual inflation (see Fig. 4a, b).

## Discussion

In this study, we aimed to test whether correction for population stratification using estimates of global ancestry adequately controls type-I error rate (at the SNP or gene-based level) and adequately retains statistical power in ethnically heterogeneous GWAS data. Towards this end, we performed simulations of population structure arising from two genetically distinct but homogenous ancestral populations as well as from the presence of samples showing recent admixture. We tested explicitly whether the degree of residual inflation depends on the degree of population divergence (F_ST_), as we hypothesized that population stratification would be more difficult to control for SNPs with relatively high values of F_ST_ .

Extensive simulations showed that correction for population stratification by including ten genomic components (MDS) sufficiently controlled inflation of type-I error rate in the CEU and YRI Hapmap populations. However, analyses of the CEU and the more strongly admixed ASW population of the 1KG data showed some inflation of type-I error rate in SNPs with F_ST_ >0.25 and SNPs with F_ST_ >0.50, with genomic inflation factors estimated at 1.02 and 1.04, respectively. Current methods (such as genomic control or principal components) for adjusting for population stratification reflect the “average SNP” in the genome and indeed generally apply a “uniform” correction (i.e., the same genomic inflation control or fixed number of genomic components across the genome), but these results suggest that SNPs with unusually strong differentiation across ancestral populations may necessitate more refined approaches in the presence of complex forms of population stratification. For example, local genomic ancestry may be taken into account in addition to a global correction for population stratification. Furthermore, such SNPs with high F_ST_ (because they may implicate loci under selection or may reflect signatures of local adaptation) may be disproportionately enriched for truly trait-associated SNPs (in comparison with selectively neutral SNPs) and should perhaps be prioritized in GWAS analysis. Our results, however, suggest that such prioritization may not be so straightforward since (a) current methods to minimize spurious associations at these SNPs may not be sufficient and (b) the degree of inflation in type-I error rate and the extent of the loss in power may depend on the nature of the ancestral heterogeneity in the samples. We note that the genomic inflation is proportional to the sample size of the study; any confounding from population stratification may be amplified by the sample size. Furthermore, linkage disequilibrium patterns resulting from admixture from previously diverged populations may contribute to an elevated genomic inflation factor (see Eq. 2). Thus, the potential confounding from population structure for large-scale studies involving admixture is a (doubly) important concern. Furthermore, while effect size estimates in the Hapmap data were largely unaffected by the correction for population stratification, the correction in the presence of admixed samples led to a reduction in statistical power. In fact, in simulations of both Hapmap and 1KG data, we observed that statistical power was reduced due to the inclusion of MDS components, but this is in part due to the relatively small sample sizes. Current GWAS sample sizes are usually at least 10 to 1000 times larger than those used in our simulations, which will diminish the effect of including 10 to 20 covariates. Nevertheless, the admixed 1KG samples showed additional loss of power from the correction for population stratification in the presence of population trait divergence.

Gene-based tests that adjust for global ancestry components sufficiently controlled type-I error rate in the admixed samples or in the mixture of two highly distinct but homogeneous populations although we did find that the required number of MDS components needed to be increased (from 10 to 20) to sufficiently account for population stratification, suggesting that stricter correction needs to be applied when multiple correlated variants are simultaneously tested. The lack of inflation stands in contrast with the results of the SNP-based analyses, which is likely explained by the fact that we did not distinguish SNPs with high F_ST_ in the gene-based tests. Such a distinction is not feasible for gene-based tests since gene-sets combine variants with different levels of F_ST_ .

Although the degree of inflation of type-I error rate in the admixed samples of the 1KG data is small, we do advice to be cautious when interpreting positive associations observed in such samples, especially if the significantly associated SNP has a high level of F_ST_. In such instances, a subset analysis in a more homogeneous population (e.g., typically of European descent) could be followed up by a test on more strongly admixed samples. Confidence in the association increases if it holds in the homogeneous subset and becomes more significant after the inclusion of the admixed samples. We would further like to stress that even though population stratification seems to be well controlled when the populations that are combined are genetically homogeneous, such as in the CEU and YRI populations, inflation may still be a problem when population structure is more complex. For example, Liu and colleagues showed that inflation is more severe in samples that include more than two divergent populations (Liu et al. 2013). The impact of the correction for population stratification in studies involving mosaic genomes from multiple ancestral populations is also warranted. Furthermore, in addition to single variant analyses and gene-based tests, Browning & Browning (Browning and Browning 2011) showed that population structure can inflate SNP-based heritability estimates derived from linear mixed models, such as implemented in GCTA (Yang et al. 2011). Incorporating principal components in linear mixed models may not be adequate and indeed may yield a substantially similar estimate as the non-adjusted model. Finally, population stratification is only one potential source of confounding that should be taken into account. Other factors that should be considered are unmeasured technical confounders (e.g., genotyping in different laboratories), selection bias and differential misclassification of exposure (Clayton et al. 2005).
